# Recontamination of Healthcare Surfaces by Repeated Wiping with Biocide-Loaded Wipes: “*One Wipe, One Surface, One Direction, Dispose*” as Best Practice in the Clinical Environment

**DOI:** 10.3390/ijms21249659

**Published:** 2020-12-18

**Authors:** Nicholas W. M. Edwards, Emma L. Best, Parikshit Goswami, Mark H. Wilcox, Stephen J. Russell

**Affiliations:** 1 School of Design, University of Leeds, Leeds LS2 9JT, UK; S.J.Russell@leeds.ac.uk; 2 Department of Microbiology, Leeds Teaching Hospitals NHS Trust, Leeds LS1 3EX, UK; emma.best@leedsth.nhs.uk (E.L.B.); mark.wilcox@nhs.net (M.H.W.); 3 Technical Textiles Research Centre, University of Huddersfield, Huddersfield HD1 3DH, UK; P.Goswami@hud.ac.uk

**Keywords:** bacteria, infection control, antimicrobial, biocides, HCAI, nonwoven, surface disinfection

## Abstract

The wiping of high-touch healthcare surfaces made of metals, ceramics and plastics to remove bacteria is an accepted tool in combatting the transmission of healthcare-associated infections (HCAIs). In practice, surfaces may be repeatedly wiped using a single wipe, and the potential for recontamination may be affected by various factors. Accordingly, we studied how the surface to be wiped, the type of fibre in the wipe and how the presence of liquid biocide affected the degree of recontamination. Experiments were conducted using metal, ceramic and plastic healthcare surfaces, and two different wipe compositions (hygroscopic and hydrophilic), with and without liquid biocide. Despite initially high removal efficiencies of >70% during initial wiping, all healthcare surfaces were recontaminated with *E. coli*, *S. aureus* and *E. faecalis* when wiped more than once using the same wipe. Recontamination occurred regardless of the fibre composition of the wipe or the presence of a liquid biocide. The extent of recontamination by *E. coli*, *S. aureus* and *E. faecalis* bacteria also increased when metal healthcare surfaces possessed a higher microscale roughness (<1 μm), as determined by Atomic Force Microscopy (AFM). The high propensity for healthcare surfaces to be re-contaminated following initial wiping suggests that a “One wipe, One surface, One direction, Dispose” policy should be implemented and rigorously enforced.

## 1. Introduction

Wipes used in combination with liquid biocides frequently form part of disinfection and decontamination regimens to remove and kill microorganisms, including pathogenic bacteria, bacterial endospores, fungi and viruses, from high-touch clinical surfaces [1]. The overall goal is to minimise the transmission of healthcare-associated infections (HCAIs) as well as the associated morbidity, mortality and financial impacts [2,3,4,5,6]. Wiping is intended to remove all bacterial contamination, as well as to prevent the transfer of wiped microorganisms from one surface to another to minimise transmission. Further information on this can be found in the work of Siani et al. [7]

The addition of an aqueous medium such as a biocide to a wipe is known to substantially improve the removal efficiency of bacteria, depending on the absorptive capacity of the nonwoven fabric, and the same is true for solid contaminants [8,9]. However, less is known about the factors affecting the degree of recontamination of surfaces due to repeated wiping. If bacteria cannot be effectively retained by the wipe during repeated wiping cycles, the mechanisms that lead to bacterial death may be compromised, depending on the antimicrobial biocides in the wipe. It is therefore important to understand the degree to which the healthcare surface itself, which is typically metal, ceramic or plastic, affects recontamination. It is also essential to minimise the risk of spreading pathogenic bacteria over a wider area and increasing the potential for HCAI transmission [1,10]. Accordingly, the pu of liquid biocide.

## 2. Results

Low-maintenance solid surfaces made of metals, ceramics and plastics are commonplace as high-touch materials in clinical settings [11,12,13,14]. The structure and chemical composition of a healthcare surface potentially influences the way in which bacteria interact and adhere [15]. Furthermore, it is known that some biocides can degrade healthcare surfaces due to prolonged or repeated exposure [16], and this wear and tear, as well as multiple cycles of cleaning and disinfection over the course of their lifetime, can affect their properties [17,18].

### 2.1. Analysis of Surfaces

**Figure 1:**
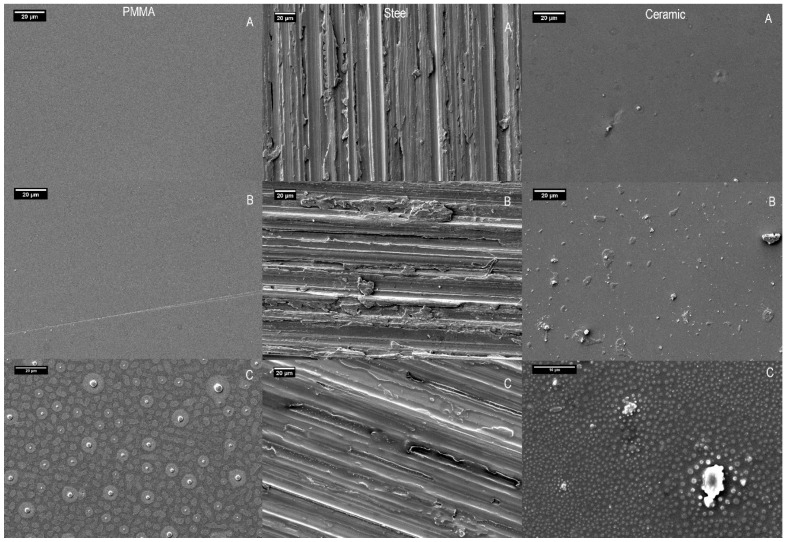
SEM images of poly (methyl methacrylate) (PMMA), steel (Rough) and ceramic surface samples. (**A**) Sterile control; (**B**) dH_2_O; (**C**) biocide.

The EDX data enabled the proportion by weight of elements present in each healthcare surface to be determined before and after wiping (Table 1). The elemental composition of the uncontaminated control surfaces and the biocide-wiped surfaces differed due to the presence of biocide residue on each of the three different healthcare surfaces after wiping (PMMA, Steel and Ceramic). SEM micrographs (Figure 1) confirmed the presence of surface deposits after the surface had been wiped with a nonwoven wipe containing the quaternary ammonium compound biocide (Figure 1c) compared to wiping with water (dH_2_O) alone (Figure 1a,b). The change in surface characteristics due to the deposit of the biocide was most noticeable on steel (Figure 1c), but was also apparent on the other substrates. One possible reason for the deposit being more evident on steel was that the “brushed” surface finish limits the ability of fluid to bead on the material surface.

### 2.2. Residual Antimicrobial Activity

The residual biocidal deposits on each healthcare surface detected by the SEM and EDX analyses (Figure 1c and Table 1) were further investigated to determine potential for residual antimicrobial activity.

Referring to Table 2, no statistically significant difference was observed in the number of bacteria present on the untreated and biocide treated surfaces (*p* < 0.05), suggesting no residual antimicrobial effect of the biocide used in this experiment in the dry state. This finding held even when the quantity of biocide on the surface was artificially high, as was the case in these experiments.

### 2.3. Bacterial Removal Efficiency and the Effect of Surface Roughness

The removal efficiency and recontamination of the healthcare surfaces by wiping were studied with reference to three common bacteria. A smooth steel surface (Table 3), with a surface roughness value (R_a_) of 128 nm, and a rougher variant of 583 nm were included to elucidate the effect of roughness on bacterial removal and recontamination.

All surfaces (metal, ceramic and polymeric) exhibited hydrophilic behaviour with contact angles of <90° when clean. Note that the water contact angle may be dependent on bacterial contamination, and so this was measured before and after surface inoculation with the simulated organic load.

The contact angle increased, and the wetting tension decreased as the organic load increased on all surfaces (Table 3). This is attributable to the proteinaceous nature of BSA and the salts in the PBS, and confirms data reported in other sources [19].

The PMMA and ceramic surfaces were extremely smooth with nanoscale roughness values of <100 nm (R_a_), compared to both steel samples, which also varied significantly between the smooth and rough variants, with R_a_ values of 128 nm and 583 nm, respectively (all *p* < 0.05).

Wiping experiments were carried out to determine the influence of surface roughness on the bacterial removal and surface recontamination using “low organic load” conditions and wipes manufactured in the laboratory. Wiping removal efficiencies in the range of 73–89% were obtained (Table 4 and Table 5), but no significant difference was observed in the removal efficiency between bacteria, wipe substrate or surface type. Therefore, over the range of values studied, roughness has no significant effect on the removal efficiency of bacteria by PP or Lyocell nonwoven wipes loaded with quaternary ammonium compound biocidal lotion. Lee et al. [20] reported similar findings, after comparing removal from “smooth” plastic and “rough” metal surfaces, finding no significant difference (*p* > 0.05) in the number of *E. coli* and *E. faecalis* CFUs.

### 2.4. Surface Recontamination during Successive Wiping Cycles

**Figure 2:**
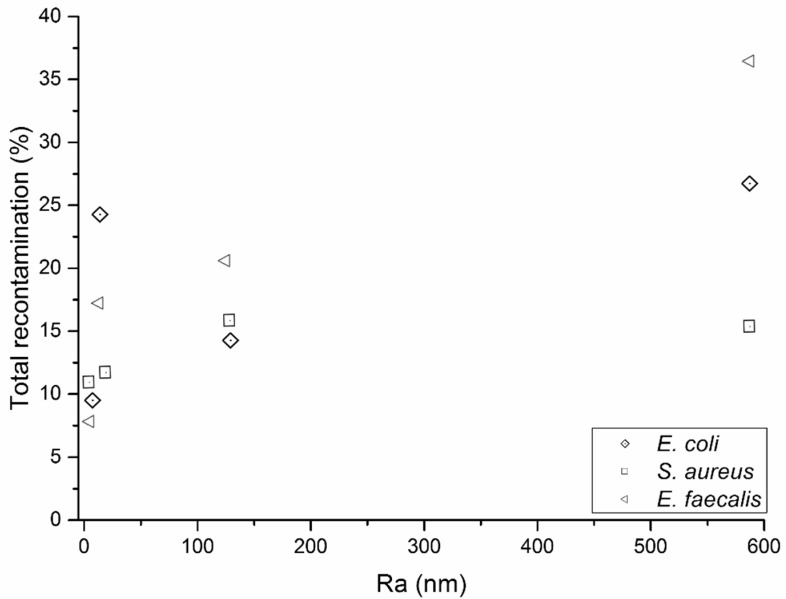
Total recontamination of surfaces by Lyocell nonwoven wipes versus the average roughness (R_a_) of the healthcare surface being wiped.

**Figure 3:**
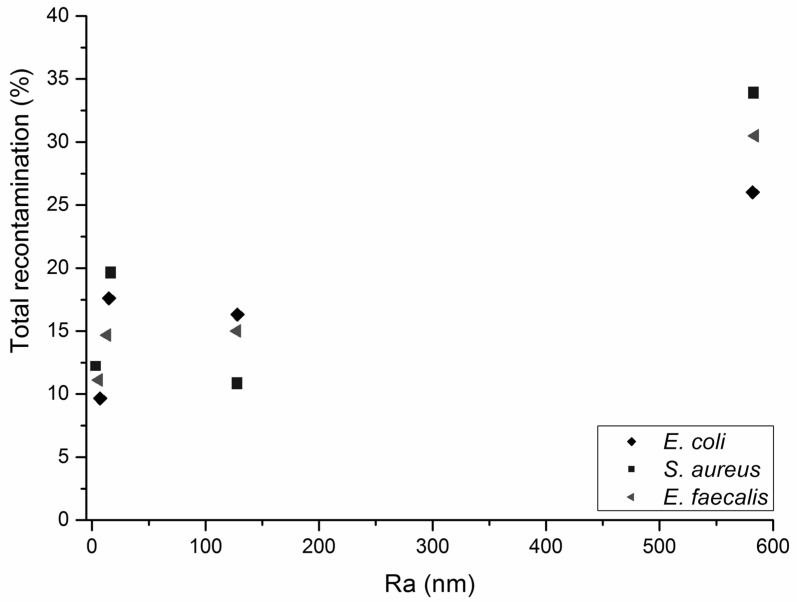
Total recontamination of surfaces by polypropylene nonwoven wipes versus average roughness (R_a_) of the healthcare surface being wiped.

During dynamic wiping, there is potential for bacteria collected by the wipe to be transferred to another area of the surface, as repeated wiping continues. The retention of the organic load is therefore an important criterion, as well as the bacterial removal efficiency. Referring to Figure 2 and Figure 3, it is evident that both wipe substrates, irrespective of their fibre composition, were unable to resist transfer of their bacterial load to uncontaminated surfaces over successive cycles.

When the surface was made of the same material, e.g., steel (smooth and rough), the proportions of bacteria transferred from the wipes after the total recontamination wiping cycles (“Total (%)”) increased with increased surface roughness (R_a_) (Table 4 and Table 5). It is therefore apparent that an increase in surface roughness, even at the nanoscale (<1 μm), is likely to increase the potential for surface recontamination during repeated wiping cycles (Figure 2 and Figure 3).

Based on these data, combined with other reported studies on the performance of commercial wipes [1,21], it is apparent that the transfer of bacteria from wipes to previously sterile surfaces is highly likely regardless of conditions. Re-contamination implies release of the bacterial load from the wipe during successive wiping cycles in a timescale that is lower than the average kill time. Note that biocidal product claims are commonly based on suspension tests, where the contact time is of the order of 5 min, rather than seconds [22,23]. In practice, a contaminated wipe containing biocide could be used multiple times across different surfaces well within the minimum 5 min timeframe needed to achieve biocidal efficacy. Given that a minimum time period of 5 min is typically needed for biocidal efficacy within the wipe, there are obvious implications in terms of the potential for the transmission of HCAIs during wiping in real healthcare environments.

Less bacteria was found to be transferred than has been reported elsewhere using detergent-loaded wipes [10] and this may be due to the specific chemical composition and more rapid kill time of the specific biocide used in the present work compared to previous studies. There is also the fact that, due to their inherent chemical composition, the cellulosic fibres in the wipes may have altered the composition of the biocidal lotion by effectively removing the cationic surfactant. By contrast, PP (and therefore the PP fibres) fibres are relatively chemically inert and should not alter the biocidal lotion. This is a promising avenue for further academic work and wipe product development.

As wiping with a typical quaternary ammonium compound biocide-loaded wipe does not appear to confer residual antimicrobial activity once applied to a target surface and leads to the recontamination of previously sterile surfaces irrespective of the roughness or chemical composition, hygiene practices should be updated to reflect this. Based on this study, and combined with other published work, regular disinfection of surfaces can be recommended with a “*One wipe, One surface, One direction, Dispose*” policy. There is a caveat to this, however, as it is possible to fold the wipe so that it can still potentially deliver a one wipe (aspect), one surface “rule”. This approach has been suggested in some healthcare settings. Wipe folding was not studied here and is a target for potential future investigation. There is the need for further work using different chemical compositions of biocide other than the quaternary ammonium compound type to see if these results can be replicated or if a given chemistry can be shown to be more effective in these situations. This should be considered alongside the “LOOK, PLAN, CLEAN AND DRY” approach reported by Dancer and Kramer [24]. Other methodologies exist for assessing bacterial transfer, including the ASTM E2967 standard [25].

## 3. Materials and Methods

### 3.1. Nonwoven Production

To minimise variations in the structure and properties of the wipes to be studied in the experimental study, and to ensure their full manufacturing history was known, samples were produced on pilot production equipment at the University of Leeds, replicating industrial nonwoven manufacturing processes. Dry-laid (carded) and hydroentangled nonwoven wipes of 100% polypropylene (PP) fibres and 100% regenerated cellulose fibres (Lyocell) were manufactured with specifications previously described by Edwards et al. [19,26].

### 3.2. Biocide and Neutraliser

#### 3.2.1. Biocide

The wipe samples were impregnated with a commercial biocide formulation used for hard surface decontamination. The biocide comprised a proprietary blend of a non-ionic surfactant (C_9_-C_11_ ethoxylated alcohol Pareth-5), a cationic surfactant (benzalkonium chloride), together with buffering agents and sequesterants. A 1:20 dilution of the biocide with deionised water (dH_2_O) led to a performance consistent with the EN 1276 “Quantitative Suspension Test of Bactericidal Activity of Chemical Disinfectants” test, giving a 5 log reduction of the pathogenic bacteria *S. aureus, E. coli, E. hirae* and *P. aeruginosa* below 5 min [27]. The diluted biocide surface tension was 37.5 × 10^−3^ N·m^−1^ at 20 °C, the viscosity was 1.35 mPa·s (60 r min^−1^ at 2.7% torque) and the pH was 9.98.

#### 3.2.2. Neutraliser Toxicity and Efficacy Tests

The neutraliser was prepared according to the method given by Ramm et al. [10]. The toxicity of the neutraliser and its ability to quench the activity of the biocide was tested according to the method reported by Knapp et al. [28].

#### 3.2.3. Impregnation of the Wipe with Biocide

Sample wipes were soaked in 10 mL 1:20 biocide or deionised water (dH_2_O-the control) for 10 min before being compressed in a Werner Mathis mangle (4 m·min^−1^) at varying pressures to achieve a liquid pickup of 150% by weight for both biocide and dH_2_O, using both the PP and the Lyocell wipes, as per Berendt et al. [29]. This 150% value was the maximum pick-up that could be achieved with the PP samples due to the hydrophobicity of the fibres, so was applied to both samples.

### 3.3. Model Healthcare Surfaces

Poly (methyl methacrylate) (PMMA) surface tiles (registered to ISO 9001), Grade 304 stainless steel (“Smooth” and “Rough” variants), and glazed ceramic tiles were selected as representative model healthcare surfaces. Where only one variant of the steel samples was tested, the Rough (R) variant was used. All surfaces were sterilised with 70% ethanol and left in ambient conditions for 10 min until visibly dry prior to use.

### 3.4. Scanning Electron Microscopy and Energy-Dispersive X-ray Spectroscopy

The chemical composition and morphology of the PMMA, “rough” steel and ceramic healthcare surfaces were analysed in the sterile state and after wiping with biocide or water (dH2O) impregnated wipe samples. Wiping was performed as described by Edwards et al. [19,26], with 10 replicates per sample. The healthcare surfaces were gold coated using a Quorum Q150RS sputter coater (Quorum Technologies Ltd.; Lewes, East Sussex, UK). A JEOL JSM-6610 LV scanning electron microscope (SEM) (JEOL Ltd.; Tokyo, Japan) was then used to image the samples, with an accelerating voltage of 5 kV, a working distance of 8 mm and a typical magnification of 750×. Energy-dispersive X-ray spectroscopy (EDX) was carried out using an Oxford Instruments INCA Xmax80 EDS Spectrometer (Oxford Instruments PLC; Abingdon, UK).

### 3.5. Surface Roughness

The surface roughness of the healthcare surfaces was analysed via atomic force microscopy (AFM). A Dimension Fastscan atomic force microscope (Bruker, Billerica, MA, USA) was used in contact dc mode to probe the surface of the steel, ceramic and PMMA under ambient conditions. Samples were mounted on a 10 mm diameter circular metal disc using epoxy resin. Nanoscope Analysis v1.5 software (Advanced Surface Microscopy, Inc., Palo Alto, CA, USA) was used to evaluate the resulting data.

### 3.6. Bacterial Strains

The microorganisms studied herein were *E. coli* (ATCC 25922), *S. aureus* (ATCC 29213) and *E. faecalis* (ATCC 29212). These were provided by Leeds Teaching Hospitals NHS Trust Pathology department (LGI; Leeds, UK). Strains were cultured according to previously published methods [19,26].

### 3.7. Microorganism Removal Efficiency from Healthcare Surfaces

The removal of bacteria from the model healthcare surfaces was evaluated according to the method described by Edwards et al. [19]. Briefly, a 900 mm^2^ section of the test fabric was attached to a 20 mm diameter boss and fixed to a Caframo BDC2002 overhead stirrer (Caframo Limited, Ontario, Canada). This was rotated at 60 r·min^−1^ for 10 s at 4.68 kN·m^−2^ applied pressure against the inoculated surface tile. The bacteria removal efficiency was calculated as in Equation (1):

R = *Cct* – *Cwt*/*Cct* × 100 (1)

where, R = removal efficiency (CFU %); *Cct* = bacterial colonies recovered from control tile; and *Cwt* = bacterial colonies recovered from wiped tile.

### 3.8. Recontamination of Surfaces

Recontamination of surfaces was measured according to the method outlined by Ramm et al. [10]. The wipe–surface contact time was 30 s at a wiping speed of 60 r·min^−1^.

The proportion of bacteria transferred was estimated based on the assumption that the difference in the number of colony-forming units (CFU) on the surface before and after wiping ended up either on or in the wipe. Owing to the nature of the recontamination calculation, statistical analysis could not be performed. This is because the total recontamination data is the sum of three consecutive transfers (T1, T2 and T3), such that T1, T2 and T3 are themselves the average transfer values for three replicates.

### 3.9. Residual Antimicrobial Activity

The assessment of residual antimicrobial activity was based on a modified Association of Official Analytical Chemists dilution method [30]. Steel, ceramic or PMMA tiles were inoculated with 20 µL of the biocide. This was spread over the surface with an L-shaped hockey stick (VWR 612-1561) using five back and forth sweeps left and right, up and down, then left and right, and allowed to dry in ambient conditions for 20 min.

Each tile was then inoculated with bacteria using the same method described by Ramm et al. [10], without simulated wiping, and assessed with a control tile (with no biocide addition). Any bacterial death on the biocide-coated surface versus the control surface was attributed to the residual antimicrobial activity of the biocide on the surface.

### 3.10. Surface Wetting Tension

The wetting behaviour of the steel, ceramic and PMMA healthcare surfaces was measured with milli-Q water using an FTÅ 1000 contact angle goniometer (First Ten Ångströms, Portsmouth, VA, USA). The healthcare surfaces were tested in the sterile state and following inoculation with 20 µL of 0.015 g·m^−2^ BSA in PBS and subsequent drying.

### 3.11. Statistical Analysis

All data are the results of at least three independent replicates. Where appropriate, a one-way analysis of variance (ANOVA) at a 95% confidence interval and a post-hoc Tukey’s test were performed. All analyses were completed in MINITAB software, version 16 (Minitab Inc.; State College, PA, USA).

## 4. Conclusions

The wiping of surfaces using a wipe that is already contaminated with bacteria during a prior wipe cycle is highly likely to result in recontamination. Regardless of the composition of the wipe (100% Lyocell or 100% PP), or the presence of a quaternary ammonium compound biocide deposit on the surface, successive wiping of a sterile surface using a wipe containing bacteria leads to recontamination. Furthermore, increasing the micro-roughness of metal healthcare surfaces increases the degree of recontamination during successive wiping cycles. No significant residual antimicrobial activity was observed following the deposition of a quaternary ammonium compound biocide on model healthcare surfaces after wiping (*p* < 0.05), confirming that there is no substantial residual biocidal activity following a wiping cycle with a quaternary ammonium compound biocide-loaded nonwoven wipe. Although quaternary ammonium compound biocide-impregnated wipes remove some of the bacterial burden from healthcare surfaces during initial wiping, it is clear that they should be used with caution since there is a risk of recontamination of otherwise uncontaminated surfaces during successive wiping. This is particularly important if the efficacy of the biocide is of the order of minutes rather than seconds.

## Figures and Tables

**Table 1:** EDX results for “Biocide” and “Control” surfaces, reporting the relative weight % of surface composition. Steel (R) denotes rough steel samples.

PMMA	Weight %		Steel (R)	Weight %		Ceramic	Weight %	
Element	Biocide	Control	Element	Biocide	Control	Element	Biocide	Control
Carbon (C)	35.71	61.88	C	32.64	7.48	C	32.47	-
Oxygen (O)	42.38	38.12	O	8.05	-	O	29.53	43.95
Sodium (Na)	14.20	-	Na	3.07	-	Na	4.59	-
Phosphorus (P)	6.34	-	P	0.98	-	Aluminium (Al)	2.70	4.26
Chlorine (Cl)	1.37	-	Cl	0.39	-	Silicon (Si)	17.34	31.97
			Chromium (Cr)	10.32	18.42	Cl	0.48	-
			Manganese (Mn)	1.3	1.65	Potassium (K)	2.48	3.84
			Iron (Fe)	39.55	65.07	Calcium (Ca)	4.36	6.74
			Nickel (Ni)	3.69	7.56	Zinc (Zn)	6.05	9.23

**Table 2:** Residual antimicrobial activity of biocide. “+” value indicates increase in bacterial recovery versus control; “−“indicates a reduction in the number of bacteria recovered versus the control. SE indicates the standard error of the mean. These values were not different from the control or each other at a statistically significant level (*p* > 0.05). Steel (R) denotes rough steel samples.

Bacteria	Surface	Mean Recovery (%)	SE
*E. coli*	Ceramic	+8	11
*S. aureus*	Ceramic	−8	3
*E. faecalis*	Ceramic	+7	13
*E. coli*	Steel (R)	−3	8
*S. aureus*	Steel (R)	−5	3
*E. faecalis*	Steel (R)	−12	9
*E. coli*	PMMA	−3	15
*S. aureus*	PMMA	−4	14
*E. faecalis*	PMMA	−16	27

**Table 3:** Contact angle, wetting tension and roughness of healthcare surfaces. Steel (R) denotes rough steel samples. Steel (S) denotes smooth steel samples. R_a_ indicates no sharing of a “grouping” letter and are significantly different-ANOVA with post hoc Tukey’s test (*p* < 0.05).

Surface	Organic Load	Contact Angle	Wetting Tension	Roughness (R_a_)	Tukey-Roughness
PMMA	Clean	29.22°	63.54 mJ·m^−2^	3.8 nm	A
	0.015 g·m^−2^ BSA	62.30°	33.84 mJ·m^−2^	n/a	
Ceramic	Clean	18.43°	69.06 mJ·m^−2^	14.8 nm	A
	0.015 g·m^−2^ BSA	38.37°	57.08 mJ·m^−2^	n/a	
Steel (S)	Clean	38.61°	65.95 mJ·m^−2^	128 nm	B
	0.015 g·m^−2^ BSA	64.20°	32.67 mJ·m^−2^	n/a	
Steel (R)	Clean	60.49°	72.80 mJ·m^−2^	583 nm	C
	0.015 g·m^−2^ BSA	63.90°	32.03 mJ·m^−2^	n/a	

**Table 4:** Removal of bacteria and recontamination of surfaces by a 100% Lyocell nonwoven wipe. Steel (R) denotes rough steel samples. Steel (S) denotes smooth steel samples.

Bacteria	Surface	Removal (%)	SE	CFU on Wipe *	T 1 (%)	T 2 (%)	T 3 (%)	Total (%)
*E. coli*	Ceramic	83	5	4,900,000	12	6	6	24
*S. aureus*	Ceramic	81	4	10,250,000	7	2	2	11
*E. faecalis*	Ceramic	73	7	2,016,667	12	3	2	17
*E. coli*	PMMA	85	7	7,700,000	8	1	1	10
*S. aureus*	PMMA	82	1	4,116,667	2	6	2	10
*E. faecalis*	PMMA	84	1	5,133,333	3	4	1	8
*E. coli*	Steel (S)	80	6	4,916,667	9	5	1	15
*S. aureus*	Steel (S)	84	4	8,350,000	8	6	2	16
*E. faecalis*	Steel (S)	87	2	3,666,667	11	9	1	21
*E. coli*	Steel (R)	83	5	4,600,000	22	2	3	27
*S. aureus*	Steel (R)	89	1	9,800,000	7	7	3	17
*E. faecalis*	Steel (R)	84	2	3,800,000	20	14	3	37

* Average number of colony-forming units on the nonwoven fabric following wiping, calculated as the difference between bacteria remaining on the surface before and after wiping [10]. T1, T2 and T3 represent the three consecutive transfers of bacteria. SE indicates the standard error of the mean.

**Table 5:** Removal of bacteria and recontamination of surfaces by a 100% polypropylene nonwoven wipe. Steel (R) denotes rough steel samples. Steel (S) denotes smooth steel samples. * Average number of colony-forming units on the nonwoven fabric following wiping, calculated as the difference between bacteria remaining on the surface before and after wiping [9]. T1, T2 and T3 represent the three consecutive transfers of bacteria. SE indicates the standard error of the mean.

Bacteria	Surface	Removal (%)	SE	CFU on Wipe *	T 1 (%)	T 2 (%)	T 3 (%)	Total (%)
*E. coli*	Ceramic	85	6	3,383,333	12	4	1	17
*S. aureus*	Ceramic	89	5	12,483,333	16	1	2	19
*E. faecalis*	Ceramic	80	2	2,483,333	11	3	1	15
*E. coli*	PMMA	79	3	9,866,667	7	2	1	10
*S. aureus*	PMMA	80	3	7,633,333	5	4	3	12
*E. faecalis*	PMMA	78	6	4,300,000	3	3	5	11
*E. coli*	Steel (S)	83	3	4,550,000	9	5	2	16
*S. aureus*	Steel (S)	83	3	8,250,000	6	4	1	11
*E. faecalis*	Steel (S)	83	6	4,700,000	10	6	1	17
*E. coli*	Steel (R)	88	1	6,600,000	11	8	7	26
*S. aureus*	Steel (R)	70	6	6,283,333	16	14	4	34
*E. faecalis*	Steel (R)	80	2	3,900,000	17	9	5	31
